# The Persisting Burden of Intracerebral Haemorrhage: Can Effective Treatments Be Found?

**DOI:** 10.1371/journal.pmed.1000353

**Published:** 2010-10-19

**Authors:** Colin B. Josephson, Joseph Frantzias, Neshika Samarasekera, Rustam Al-Shahi Salman

**Affiliations:** Division of Clinical Neurosciences, Centre for Clinical Brain Sciences, University of Edinburgh, Western General Hospital, Edinburgh, United Kingdom

## Abstract

Colin Josephson, Rustam Al-Shahi Salman, and colleagues discuss the effectiveness of treatments for intracerebral haemorrhage.

**Summary**

Intracerebral haemorrhage (ICH) accounts for ∼10% and ∼20% of strokes in high and low-middle income countries, respectively, but ICH incidence and case fatality do not appear to be declining. Evidence supports organised stroke unit care and secondary prevention with blood pressure lowering after ICH. Ongoing randomised controlled trials of treatments that are either intended to limit early ICH growth, reduce perihaematomal oedema, or modify other key pathophysiological mechanisms underlying deterioration after acute ICH, offer hope for future improvements in outcome.

## Introduction

Spontaneous intracerebral haemorrhage (ICH) that is apparently unrelated to trauma or an underlying vascular, neoplastic, or coagulopathic cause has incurred the same global burden over the past quarter of a century [1],[2]. During the last decade, spontaneous ICH accounted for ∼10% of strokes in high income countries and ∼20% of strokes in low and middle income countries, where the one month case fatalities were 25%–35% and 30%–48%, respectively [3].

The incidence of ICH is higher in Asians [2], and the major risk factors for spontaneous ICH without an identified cause (so-called primary ICH) are male gender, systemic arterial hypertension, excessive alcohol consumption, increasing age, smoking, and diabetes mellitus [4]. However, over the past quarter of a century, the incidence of primary ICH associated with pre-stroke hypertension seems to have declined, whereas there seems to have been an increase associated with antithrombotic use and presumed cerebral amyloid angiopathy in those aged ≥75 years [1]. Whilst primary prevention with antihypertensive medication is probably the most effective strategy to reduce the burden of ICH, could the management of ICH influence outcome?

The outcome after primary ICH seems to be worse than after a bleed secondary to an arteriovenous malformation [5], which justifies thorough investigation for all patients (Table 1). However, there is a shortage of evidence and lack of consensus about who, when, and how to further investigate for a cause underlying ICH [6]. There appears to be a modest association between ICH deep in the brain and hypertension, and between ICH in the lobes of the brain and cerebral amyloid angiopathy [7],[8], but these associations by no means rule out the need for further investigation of patients who are likely to survive and benefit from the identification of a treatable underlying cause (Table 1) [9].

**Table 1:** Investigations into Common Causes of Secondary Intracerebral Haemorrhage (ICH).

Investigation	Common Causes Identified by the Investigation
Further history from patient and others	Undisclosed trauma, drug use
Routine laboratory tests^a^	Vasculitis
	Liver cirrhosis
	Neoplasm (secondary)
	Infective endocarditis
Coagulation studies	Warfarin
	Von Willebrand disease
	Vitamin K deficiency
	Factor VIII, IX, XIII deficiency
Blood cultures	Infective endocarditis
Toxicology	Cocaine
	Amphetamines
Human chorionic gonadotrophin (β-hCG)	Pregnancy
Cerebrospinal fluid analysis^b^	Vasculitis
CT, CT angiography, CT venography	Neoplasm (primary or secondary)
	Intracranial arterial aneurysm
	Intracranial arteriovenous malformation
	Intracranial venous thrombosis
MRI, MR angiography, MR venography	Cavernous malformation
	Intracranial arterial aneurysm
	Intracranial arteriovenous malformation
	Haemorrhagic transformation of a cerebral infarction
	Neoplasm (primary or secondary)
Cerebral catheter angiography	Intracranial arterial aneurysm
	Intracranial arteriovenous malformation
	Dural arteriovenous fistula
	Vasculitis
Neuroradiologist review of brain imaging	All causes whose identification depends on brain imaging

a Full blood count, electrolytes, creatinine, urea, liver function tests, inflammatory markers (ESR/CRP), electrocardiogram, chest radiograph.

b Consider the risk of transtentorial herniation before undertaking.

CT, computed tomography; MRI, magnetic resonance imaging.

Apart from identifying and treating underlying causes of ICH, this review focuses on other strategies to improve outcome, bearing in mind the pathophysiological mechanisms underlying clinical deterioration after ICH. We go on to address the treatments for primary ICH that are supported by randomised controlled trials (RCTs) and those that are not, and discuss which interventions appear to be the most promising in ongoing and future RCTs.

## Pathophysiology of Acute ICH

**Figure 1:**
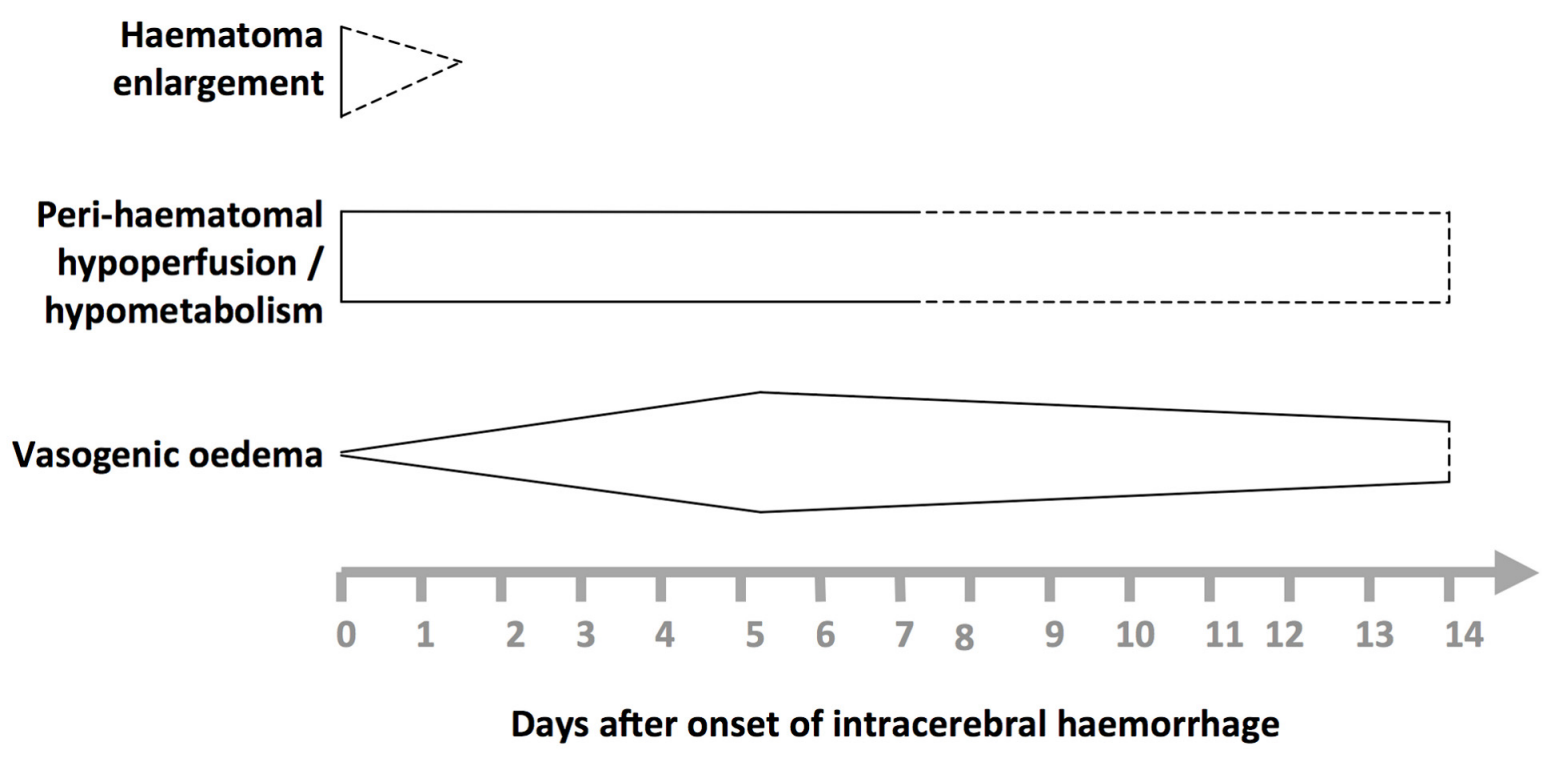
Selected pathophysiological mechanisms that have been identified in humans after acute, spontaneous intracerebral haemorrhage. Shapes are approximate illustrations of when pathophysiological mechanisms are at their peak and their known durations. Uncertainties about the duration and intensity of mechanisms are indicated by dashed lines.

**Figure 2:**
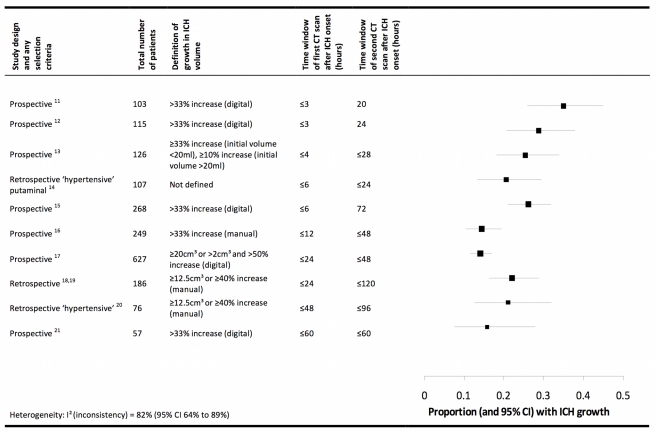
Summary of selected radiological studies of spontaneous intracerebral haemorrhage growth. Studies are organised in ascending order of the duration of the time window of the first computed tomogram. Manual calculations of haematoma volume used the ABC/2 method. We excluded data on patients taking anticoagulant drugs in these studies, and excluded studies from which data could not be extracted [77],[78], studies that incorporated patients already included in the summary above [79],[80], or studies in which interventions may have influenced haematoma growth [58],[81].

In humans, known pathophysiological mechanisms underlying further clinical deterioration soon after ICH include hydrocephalus, intraventricular extension of ICH, and recurrent ICH [10]; pathological and radiological studies have illuminated additional mechanisms (Figure 1). Human studies performing brain computed tomography within two time windows after ICH onset have documented haematoma expansion (Figure 2)—either due to growth of the original haemorrhage or re-bleeding [11]–[21]—that is associated with poor outcome [12],[16],[18],[22]. Imaging studies have demonstrated peri-haematomal hypoperfusion within the first week of ICH onset [23],[24], but not an “ischaemic penumbra” [25],[26]. However, there is evidence of a compensatory reduction in the metabolic rate, or a “metabolic penumbra”, around ICH [25],, as well as peri-haematomal hyperglycolysis (possibly due to inflammation, excitotoxicity, spreading depression, or seizures) [27]. Perihaematomal oedema appears to be vasogenic (plasma-derived) [28], its volume may increase within 24 hours of ICH onset and peak within 14 days [29]–[31], and it may be caused or exacerbated by thrombin and activated platelets [32],[33].

Animal models support the contributions to peri-haematomal oedema made by clot retraction, hydrostatic pressure, enhanced thrombin production, increased blood–brain barrier permeability, and products of erythrocyte lysis (such as haeme oxygenase-mediated liberation of iron from haeme rings) [32],[34]–[37]. ICH in animal models seems to trigger humoral and cellular inflammatory responses: the consequent migration and recruitment of neutrophils and activation of native microglia results in oxidative stress and neuronal necrosis [38],[39], cytokines such as tumour necrosis factor α and interleukin 1-β lead to apoptosis [40],[41], complement activation causes erythrocyte lysis [42], and matrix metalloproteinases may result in oedema, necrosis, and blood–brain barrier disruption [43].

A better understanding of the pathophysiology of ICH could emerge if the decline in human autopsy rates reverses [44], and if animal models of ICH better represent human ICH [45]. Rodent models of ICH involve either stereotactic intraparenchymal infusion of autologous whole blood (which may cause simultaneous intraventricular or subarachnoid haemorrhage [46]), or injection of proteolytic bacterial collagenase (which incites a vigorous immune response in excess of that seen in humans [46]), after which very few animals die, which is quite unlike spontaneous ICH in humans [2].

## Treatments Shown to Be Beneficial in Humans, Either in a Meta-Analysis of RCTs or in a Single Large RCT

A Cochrane meta-analysis of 31 RCTs involving 6,936 participants showed that organised inpatient care in stroke units benefits patients with stroke (whether ischaemic or due to ICH) by reducing the odds of death or dependency by 18% [47]. Large observational studies corroborate these findings in patients with ICH [48],[49]. Which aspects of organized stroke care, either individually or together, improve outcome in patients with ICH remain to be determined. One small, non-randomised, observational analysis, which was adjusted for some of the known influences on ICH prognosis, found survival after ICH to be better when managed in neuro-intensive care units compared to general intensive care units [50]: RCTs of some of the interventions used in the “black box” of neuro-intensive care (such as acute blood pressure lowering) are underway (see below). Furthermore, the benefits of standard care can be inferred from the effects on clinical outcome of do-not-resuscitate orders in a case-mix adjusted multi-hospital observational study [51], and withdrawal of care in a multivariable analysis at a single hospital [52].

A Cochrane meta-analysis of ten RCTs involving 2,059 participants found a reduction in death or dependence from the neurosurgical evacuation of spontaneous supratentorial ICH (odds ratio [OR] 0.71, 95% confidence interval [CI] 0.58 to 0.88) [53]. However, most of the RCTs included in this meta-analysis were of modest quality, their methods differed, and the largest RCT (Surgical Trial in Intracerebral Hemorrhage [STICH]) found no difference between early surgery or initial conservative management [54]. A sub-group with lobar ICH within 1 cm of the cortical surface appeared to benefit from surgery in STICH, so the STICH II RCT (ISRCTN 22153967) is evaluating early ICH evacuation in this sub-group of patients.

Secondary prevention with anti-hypertensive drugs reduced the risk of vascular events after stroke in the PROGRESS RCT; amongst the subset of 611 participants with ICH, the risk of subsequent stroke was halved by a perindopril-based blood pressure lowering regimen [55].

## Treatments Neither Shown to be Beneficial to Humans in a Meta-Analysis of RCTs, Nor in a Single Large RCT

Haemostatic drugs are a biologically plausible intervention to improve outcome after ICH by limiting the early growth of spontaneous ICH (Figure 2). Despite the ability of recombinant activated factor VII (rFVIIa) to curtail early haematoma growth by 4–6 ml, a Cochrane meta-analysis of four RCTs involving 1,305 participants found that this surrogate outcome did not translate into any net clinical benefit (risk ratio of death or dependence [modified Rankin Scale score 4 to 6] at 90 days = 0.91 [95% CI 0.72 to 1.15]). This reduction in ICH growth may have been too small to improve clinical outcome, its benefit may have been offset by the thrombo-embolic adverse effects of rFVIIa, or the RCTs might have been unable to detect a small benefit of rFVIIa because of insufficient precision or some methodological weaknesses [56]. Other haemostatic drugs including antifibrinolytic agents seem to be worth testing in future RCTs.

Similarly, early blood pressure lowering might improve outcome after ICH by limiting the early growth of spontaneous ICH, but there has been a shortage of evidence supporting this intervention, unsurprisingly leading to differences between ICH guidelines [9],[57]. The Intensive Blood Pressure Reduction in Acute Cerebral Haemorrhage Trial (INTERACT) randomized 404 patients presenting within 6 hours of onset to a systolic blood pressure target of ≤140 mmHg achieved within 1 hour and continued for 7 days, versus the American Heart Association guideline's target [9],[58]. There was a non-significant reduction in ICH growth by 1–2 ml, and no effect on clinical outcome, but the safety data are encouraging for the large, ongoing Intensive Blood Pressure Reduction in Acute Cerebral Haemorrhage Trial (INTERACT-2; ISRCTN 73916115).

Attenuating peri-haematomal oedema might improve outcome after ICH (Figure 1), but meta-analyses have demonstrated neither benefit nor harm from dexamethasone (five RCTs involving 206 participants) [59], glycerol (two RCTs involving 224 participants) [60], and mannitol (two RCTs involving 149 participants) [61]. Neuroprotection, too, has caused neither benefit nor harm after acute ICH with the anti-oxidant free-radical scavenger NXY-059 (one RCT involving 607 participants) [62] or the glycine antagonist gavestinel (one RCT involving 571 participants) [63].

## Future Directions

### Attenuation of Haematoma Growth

The major treatment target of ongoing RCTs is haematoma growth, because ICH size and growth are determinants of outcome [12],. So far, the attenuation of early ICH growth seen with the haemostatic agent rFVIIa [56] or intensive acute blood pressure lowering [58],[64] has not improved clinical outcome in the RCTs performed. However, this biologically plausible mechanism for improving outcome is worthy of further research in RCTs that are large enough to detect small clinical benefits, such as the ongoing RCTs of acute blood pressure lowering (including INTERACT-2, the Efficacy of Nitric Oxide in Stroke Trial [ENOS; ISRCTN 99414122], Antihypertensive Treatment of Acute Cerebral Hemorrhage II [ATACH-II; ISRCTN R01-NS044976], and the Scandinavian Candesartan Acute Stroke Trial [SCAST; ISRCTN 13643354]). RCTs of alternative approaches to improve outcome after primary ICH by limiting haematoma expansion include rFVIIa in sub-groups of patients whose haematomas are more likely to grow (The Spot Sign for Predicting and Treating ICH Growth Study [STOP-IT; NCT00810888]), or testing the effectiveness of antifibrinolytic drugs such as the lysine analogue tranexamic acid, which seems to have attenuated ICH growth in two non-randomised studies [65],[66].

The greater risk of haematoma expansion and death after ICH associated with warfarin [67] and the contemporary increase in the incidence of primary ICH associated with all antithrombotic drugs [1] make RCTs of the management of antithrombotic-associated ICH a priority. Whilst stopping warfarin after ICH is common sense, and intravenous vitamin K administration is standard practice, there is a shortage of evidence about how else to treat anticoagulant-associated ICH [68], so RCTs comparing prothrombin complex concentrate with fresh frozen plasma in this context are ongoing (International Normalized Ratio (INR) Normalization in Coumadin Associated Intracerebral Haemorrhage [INCH; NCT00928915] and Efficacy and Safety of BERIPLEX P/N Compared with Plasma in Patients with Acute Major Bleeding Caused by Anticoagulant Therapy [NCT00708435]), as are studies of rFVIIa. The finding that mortality is higher for patients who were on antiplatelet agents at the time of ICH compared to those who were not [69] has led to an ongoing RCT of platelet transfusion to limit ICH growth and improve outcome after ICH associated with antiplatelet drugs (Platelet Transfusion in Cerebral Haemorrhage [PATCH; http://www.strokecenter.org/trials/TrialDetail.aspx?tid=730).

### Other Approaches

Firstly, targeting other potentially treatable determinants of poor outcome after ICH may be fruitful. Intraventricular extension of ICH is one such mechanism [10], and there are two RCTs of ventricular drainage combined with intraventricular recombinant tissue plasminogen activator (Dutch Intraventricular Thrombolysis after Cerebral Haemorrhage Study [DITCH; ISRCTN 19105863] and Clot Lysis: Evaluation Acceleration of Resolution of IVH [CLEAR-IVH; NCT00650858]).

Secondly, just as the PATCH RCT is a response to the apparent rise in incidence of antiplatelet-associated ICH, the apparent rise in the incidence of lobar ICH that may be caused by cerebral amyloid angiopathy merits consideration of treatments that might reduce amyloid deposition [1]. Tramiprosate is a synthetic compound that competes with glycosaminoglycans for binding to β-amyloid peptide, reducing amyloid fibril formation and deposition, and demonstrated a good safety profile in a phase II study [70]. Amyloid-depleting agents, which have shown remarkable effects in Alzheimer's disease [71], are an alternative approach and may be preferable to amyloid-β immunisation, which can induce an immune-mediated encephalomyelitis [72].

Lastly, treatments that have proven beneficial in animal models might translate from the bench to the bedside, although there are concerns about the rodent ICH models used and the methodological quality of animal experiments [45],[46],[73]. One such example is deferoxamine (an iron-chelating agent that crosses the blood–brain barrier, and has been associated with a reduction in brain oedema, neurological deficits, and biochemical markers of oxidative damage in animals) [74],[75], which has led to the Dose Finding and Safety study of Deferoxamine in Patients with Brain Hemorrhage (DFO in ICH; NCT00598572).

## Conclusions

The incidence and risk of dying from ICH seem not to have changed in recent decades, whilst the incidence of ischaemic stroke has declined [2],[3]. In contrast to the advances in the treatment of ischaemic stroke, stroke unit care and secondary prevention with blood pressure reduction are the only interventions for patients with stroke due to ICH that are based on robust evidence [47],[55]. However, insights gleaned from radiological and pathological investigations of the cause and pathophysiology of ICH, and the relentless pursuit of potential treatments in ongoing RCTs, are all cause for optimism [76].

**Five Key Papers in the Field**

- van Asch CJ, Luitse MJ, Rinkel GJ, van der Tweel I, Algra A, et al. (2010) Incidence, case fatality, and functional outcome of intracerebral haemorrhage over time, according to age, sex, and ethnic origin: a systematic review and meta-analysis. Lancet Neurol 9(2): 167–176.
- Davis SM, Broderick J, Hennerici M, Brun NC, Diringer MN, et al. (2006) Hematoma growth is a determinant of mortality and poor outcome after intracerebral hemorrhage. Neurology 66(8): 1175–1181.
- Stroke Unit Trialists' Collaboration (2007) Organised inpatient (stroke unit) care for stroke. Cochrane Database Syst Rev (4): CD000197.
- Chapman N, Huxley R, Anderson C, Bousser MG, Chalmers J, et al. (2004) Effects of a perindopril-based blood pressure-lowering regimen on the risk of recurrent stroke according to stroke subtype and medical history: the PROGRESS Trial. Stroke 35(1): 116–121.
- NINDS ICH Workshop Participants (2005) Priorities for clinical research in intracerebral hemorrhage: report from a National Institute of Neurological Disorders and Stroke workshop. Stroke 36(3): e23–e41.

## Abbreviations

95% CI: 95% confidence interval
ICH: intracerebral haemorrhage
OR: odds ratio
RCT: randomised controlled trial

## References

[1] Lovelock CE, Molyneux AJ, Rothwell PM (2007). Change in incidence and aetiology of intracerebral haemorrhage in Oxfordshire, UK, between 1981 and 2006: a population-based study.. Lancet Neurol.

[2] van Asch CJ, Luitse MJ, Rinkel GJ, van der Tweel I, Algra A (2010). Incidence, case fatality, and functional outcome of intracerebral haemorrhage over time, according to age, sex, and ethnic origin: a systematic review and meta-analysis.. Lancet Neurol.

[3] Feigin VL, Lawes CM, Bennett DA, Barker-Collo SL, Parag V (2009). Worldwide stroke incidence and early case fatality reported in 56 population-based studies: a systematic review.. Lancet Neurol.

[4] Ariesen MJ, Claus SP, Rinkel GJ, Algra A (2003). Risk factors for intracerebral hemorrhage in the general population: a systematic review.. Stroke.

[5] van Beijnum J, Lovelock CE, Cordonnier C, Rothwell PM, Klijn CJ (2009). Outcome after spontaneous and arteriovenous malformation-related intracerebral haemorrhage: population-based studies.. Brain.

[6] Cordonnier C, Klijn CJ, van Beijnum J, Al-Shahi Salman R (2010). Radiological investigation of spontaneous intracerebral hemorrhage: systematic review and trinational survey.. Stroke.

[7] Ritter MA, Droste DW, Hegedus K, Szepesi R, Nabavi DG (2005). Role of cerebral amyloid angiopathy in intracerebral hemorrhage in hypertensive patients.. Neurology.

[8] Jackson CA, Sudlow CL (2006). Is hypertension a more frequent risk factor for deep than for lobar supratentorial intracerebral haemorrhage?. J Neurol Neurosurg Psychiatry.

[9] Broderick J, Connolly S, Feldmann E, Hanley D, Kase C (2007). Guidelines for the management of spontaneous intracerebral hemorrhage in adults: 2007 update: a guideline from the American Heart Association/American Stroke Association Stroke Council, High Blood Pressure Research Council, and the Quality of Care and Outcomes in Research Interdisciplinary Working Group.. Stroke.

[10] Qureshi AI, Mendelow AD, Hanley DF (2009). Intracerebral haemorrhage.. Lancet.

[11] Brott T, Broderick J, Kothari R, Barsan W, Tomsick T (1997). Early hemorrhage growth in patients with intracerebral hemorrhage.. Stroke.

[12] Davis SM, Broderick J, Hennerici M, Brun NC, Diringer MN (2006). Hematoma growth is a determinant of mortality and poor outcome after intracerebral hemorrhage.. Neurology.

[13] Ji N, Lu JJ, Zhao YL, Wang S, Zhao JZ (2009). Imaging and clinical prognostic indicators for early hematoma enlargement after spontaneous intracerebral hemorrhage.. Neurol Res.

[14] Fujitsu K, Muramoto M, Ikeda Y, Inada Y, Kim I (1990). Indications for surgical treatment of putaminal hemorrhage. Comparative study based on serial CT and time-course analysis.. J Neurosurg.

[15] Sansing LH, Messe SR, Cucchiara BL, Cohen SN, Lyden PD (2009). Prior antiplatelet use does not affect hemorrhage growth or outcome after ICH.. Neurology.

[16] Leira R, Davalos A, Silva Y, Gil-Peralta A, Tejada J (2004). Early neurologic deterioration in intracerebral hemorrhage: predictors and associated factors.. Neurology.

[17] Fujii Y, Takeuchi S, Sasaki O, Minakawa T, Tanaka R (1998). Multivariate analysis of predictors of hematoma enlargement in spontaneous intracerebral hemorrhage.. Stroke.

[18] Kazui S, Naritomi H, Yamamoto H, Sawada T, Yamaguchi T (1996). Enlargement of spontaneous intracerebral hemorrhage. Incidence and time course.. Stroke.

[19] Kazui S, Minematsu K, Yamamoto H, Sawada T, Yamaguchi T (1997). Predisposing factors to enlargement of spontaneous intracerebral hematoma.. Stroke.

[20] Ohwaki K, Yano E, Nagashima H, Hirata M, Nakagomi T (2004). Blood pressure management in acute intracerebral hemorrhage: relationship between elevated blood pressure and hematoma enlargement.. Stroke.

[21] Flibotte JJ, Hagan N, O'Donnell J, Greenberg SM, Rosand J (2004). Warfarin, hematoma expansion, and outcome of intracerebral hemorrhage.. Neurology.

[22] Hemphill JC, Bonovich DC, Besmertis L, Manley GT, Johnston SC (2001). The ICH score: a simple, reliable grading scale for intracerebral hemorrhage.. Stroke.

[23] Rosand J, Eskey C, Chang Y, Gonzalez RG, Greenberg SM (2002). Dynamic single-section CT demonstrates reduced cerebral blood flow in acute intracerebral hemorrhage.. Cerebrovasc Dis.

[24] Pascual AM, Lopez-Mut JV, Benlloch V, Chamarro R, Soler J (2007). Perfusion-weighted magnetic resonance imaging in acute intracerebral hemorrhage at baseline and during the 1st and 2nd week: a longitudinal study.. Cerebrovasc Dis.

[25] Zazulia AR, Diringer MN, Videen TO, Adams RE, Yundt K (2001). Hypoperfusion without ischemia surrounding acute intracerebral hemorrhage.. J Cereb Blood Flow Metab.

[26] Herweh C, Juttler E, Schellinger PD, Klotz E, Jenetzky E (2007). Evidence against a perihemorrhagic penumbra provided by perfusion computed tomography.. Stroke.

[27] Zazulia AR, Videen TO, Powers WJ (2009). Transient focal increase in perihematomal glucose metabolism after acute human intracerebral hemorrhage.. Stroke.

[28] Butcher KS, Baird T, MacGregor L, Desmond P, Tress B (2004). Perihematomal edema in primary intracerebral hemorrhage is plasma derived.. Stroke.

[29] Zazulia AR, Diringer MN, Derdeyn CP, Powers WJ (1999). Progression of mass effect after intracerebral hemorrhage.. Stroke.

[30] Gebel JM, Jauch EC, Brott TG, Khoury J, Sauerbeck L (2002). Natural history of perihematomal edema in patients with hyperacute spontaneous intracerebral hemorrhage.. Stroke.

[31] Inaji M, Tomita H, Tone O, Tamaki M, Suzuki R (2003). Chronological changes of perihematomal edema of human intracerebral hematoma.. Acta Neurochir Suppl.

[32] Gebel JM, Brott TG, Sila CA, Tomsick TA, Jauch E (2000). Decreased perihematomal edema in thrombolysis-related intracerebral hemorrhage compared with spontaneous intracerebral hemorrhage.. Stroke.

[33] Sansing LH, Kaznatcheeva EA, Perkins CJ, Komaroff E, Gutman FB (2003). Edema after intracerebral hemorrhage: correlations with coagulation parameters and treatment.. J Neurosurg.

[34] Wagner KR, Xi G, Hua Y, Kleinholz M, de Court (1996). Lobar intracerebral hemorrhage model in pigs: rapid edema development in perihematomal white matter.. Stroke.

[35] Lee KR, Kawai N, Kim S, Sagher O, Hoff JT (1997). Mechanisms of edema formation after intracerebral hemorrhage: effects of thrombin on cerebral blood flow, blood-brain barrier permeability, and cell survival in a rat model.. J Neurosurg.

[36] Huang FP, Xi G, Keep RF, Hua Y, Nemoianu A (2002). Brain edema after experimental intracerebral hemorrhage: role of hemoglobin degradation products.. J Neurosurg.

[37] Levine JM, Snider R, Finkelstein D, Gurol ME, Chanderraj R (2007). Early edema in warfarin-related intracerebral hemorrhage.. Neurocrit Care.

[38] Weiss SJ (1989). Tissue destruction by neutrophils.. N Engl J Med.

[39] Power C, Henry S, Del Bigio MR, Larsen PH, Corbett D (2003). Intracerebral hemorrhage induces macrophage activation and matrix metalloproteinases.. Ann Neurol.

[40] Mayne M, Ni W, Yan HJ, Xue M, Johnston JB (2001). Antisense oligodeoxynucleotide inhibition of tumor necrosis factor-alpha expression is neuroprotective after intracerebral hemorrhage.. Stroke.

[41] Holmin S, Mathiesen T (2000). Intracerebral administration of interleukin-1beta and induction of inflammation, apoptosis, and vasogenic edema.. J Neurosurg.

[42] Xi G, Hua Y, Keep RF, Younger JG, Hoff JT (2001). Systemic complement depletion diminishes perihematomal brain edema in rats.. Stroke.

[43] Wang J, Tsirka SE (2005). Neuroprotection by inhibition of matrix metalloproteinases in a mouse model of intracerebral haemorrhage.. Brain.

[44] Ayoub T, Chow J (2008). The conventional autopsy in modern medicine.. J R Soc Med.

[45] James ML, Warner DS, Laskowitz DT (2008). Preclinical models of intracerebral hemorrhage: a translational perspective.. Neurocrit Care.

[46] Andaluz N, Zuccarello M, Wagner KR (2002). Experimental animal models of intracerebral hemorrhage.. Neurosurg Clin N Am.

[47] Stroke Unit Trialists' Collaboration (2007). Organised inpatient (stroke unit) care for stroke.. Cochrane Database Syst Rev.

[48] Candelise L, Gattinoni M, Bersano A, Micieli G, Sterzi R (2007). Stroke-unit care for acute stroke patients: an observational follow-up study.. Lancet.

[49] Terent A, Asplund K, Farahmand B, Henriksson KM, Norrving B (2009). Stroke unit care revisited: who benefits the most? A cohort study of 105,043 patients in Riks-Stroke, the Swedish Stroke Register.. J Neurol Neurosurg Psychiatry.

[50] Diringer MN, Edwards DF (2001). Admission to a neurologic/neurosurgical intensive care unit is associated with reduced mortality rate after intracerebral hemorrhage.. Crit Care Med.

[51] Hemphill JC, Newman J, Zhao S, Johnston SC (2004). Hospital usage of early do-not-resuscitate orders and outcome after intracerebral hemorrhage.. Stroke.

[52] Becker KJ, Baxter AB, Cohen WA, Bybee HM, Tirschwell DL (2001). Withdrawal of support in intracerebral hemorrhage may lead to self-fulfilling prophecies.. Neurology.

[53] Prasad K, Mendelow AD, Gregson B (2008). Surgery for primary supratentorial intracerebral haemorrhage.. Cochrane Database Syst Rev.

[54] Mendelow AD, Gregson BA, Fernandes HM, Murray GD, Teasdale GM (2005). Early surgery versus initial conservative treatment in patients with spontaneous supratentorial intracerebral haematomas in the International Surgical Trial in Intracerebral Haemorrhage (STICH): a randomised trial.. Lancet.

[55] Chapman N, Huxley R, Anderson C, Bousser MG, Chalmers J (2004). Effects of a perindopril-based blood pressure-lowering regimen on the risk of recurrent stroke according to stroke subtype and medical history: the PROGRESS Trial.. Stroke.

[56] Al-Shahi Salman R (2009). Haemostatic drug therapies for acute spontaneous intracerebral haemorrhage.. Cochrane Database Syst Rev.

[57] Steiner T, Kaste M, Forsting M, Mendelow D, Kwiecinski H (2006). Recommendations for the management of intracranial haemorrhage - part I: spontaneous intracerebral haemorrhage. The European Stroke Initiative Writing Committee and the Writing Committee for the EUSI Executive Committee.. Cerebrovasc Dis.

[58] Anderson CS, Huang Y, Wang JG, Arima H, Neal B (2008). Intensive blood pressure reduction in acute cerebral haemorrhage trial (INTERACT): a randomised pilot trial.. Lancet Neurol.

[59] Feigin VL, Anderson N, Rinkel GJ, Algra A, van GJ (2005). Corticosteroids for aneurysmal subarachnoid haemorrhage and primary intracerebral haemorrhage.. Cochrane Database Syst Rev.

[60] Righetti E, Celani MG, Cantisani T, Sterzi R, Boysen G (2004). Glycerol for acute stroke.. Cochrane Database Syst Rev.

[61] Bereczki D, Fekete I, Prado GF, Liu M (2007). Mannitol for acute stroke.. Cochrane Database Syst Rev.

[62] Lyden PD, Shuaib A, Lees KR, Davalos A, Davis SM (2007). Safety and tolerability of NXY-059 for acute intracerebral hemorrhage: the CHANT Trial.. Stroke.

[63] Haley EC, Thompson JL, Levin B, Davis S, Lees KR (2005). Gavestinel does not improve outcome after acute intracerebral hemorrhage: an analysis from the GAIN International and GAIN Americas studies.. Stroke.

[64] Geeganage C, Bath PM (2008). Interventions for deliberately altering blood pressure in acute stroke.. Cochrane Database Syst Rev.

[65] Sorimachi T, Fujii Y, Morita K, Tanaka R (2005). Rapid administration of antifibrinolytics and strict blood pressure control for intracerebral hemorrhage.. Neurosurgery.

[66] Ojacastro MF, Tabuena MP, Dulos ID, Tabuena RP (2008). Efficacy of tranexamic acid in reducing hematoma volume in patients with hypertensive intracerebral hemorrhage.. Int J Stroke.

[67] Cucchiara B, Messe S, Sansing L, Kasner S, Lyden P (2008). Hematoma growth in oral anticoagulant related intracerebral hemorrhage.. Stroke.

[68] Aguilar MI, Hart RG, Kase CS, Freeman WD, Hoeben BJ (2007). Treatment of warfarin-associated intracerebral hemorrhage: literature review and expert opinion.. Mayo Clin Proc.

[69] Thompson BB, Bejot Y, Caso V, Castillo J, Christensen H (2010). Prior antiplatelet therapy and outcome following intracerebral hemorrhage. A systematic review.. Neurology.

[70] Greenberg SM, Rosand J, Schneider AT, Creed PL, Gandy SE (2006). A phase 2 study of tramiprosate for cerebral amyloid angiopathy.. Alzheimer Dis Assoc Disord.

[71] Kolstoe SE, Ridha BH, Bellotti V, Wang N, Robinson CV (2009). Molecular dissection of Alzheimer's disease neuropathology by depletion of serum amyloid P component.. Proc Natl Acad Sci U S A.

[72] Orgogozo JM, Gilman S, Dartigues JF, Laurent B, Puel M (2003). Subacute meningoencephalitis in a subset of patients with AD after Abeta42 immunization.. Neurology.

[73] Frantzias J, Sena ES, Macleod MR, Al-Shahi Salman R (2010). Treatment of intracerebral hemorrhage in animal models: Meta-analysis. Ann Neurol.

[74] Nakamura T, Keep RF, Hua Y, Schallert T, Hoff JT (2004). Deferoxamine-induced attenuation of brain edema and neurological deficits in a rat model of intracerebral hemorrhage.. J Neurosurg.

[75] Gu Y, Hua Y, Keep RF, Morgenstern LB, Xi G (2009). Deferoxamine reduces intracerebral hematoma-induced iron accumulation and neuronal death in piglets.. Stroke.

[76] NINDS ICH Workshop Participants (2005). Priorities for clinical research in intracerebral hemorrhage: report from a National Institute of Neurological Disorders and Stroke workshop.. Stroke.

[77] Herold S, von KR, Jaeger C (1982). Follow-up of spontaneous intracerebral haemorrhage by computed tomography.. J Neurol.

[78] Kelley RE, Berger JR, Scheinberg P, Stokes N (1982). Active bleeding in hypertensive intracerebral hemorrhage: computed tomography.. Neurology.

[79] Silva Y, Leira R, Tejada J, Lainez JM, Castillo J (2005). Molecular signatures of vascular injury are associated with early growth of intracerebral hemorrhage.. Stroke.

[80] Jauch EC, Lindsell CJ, Adeoye O, Khoury J, Barsan W (2006). Lack of evidence for an association between hemodynamic variables and hematoma growth in spontaneous intracerebral hemorrhage.. Stroke.

[81] Qureshi AI, Palesch YY, Martin R, Novitzke J, Cruz-Flores S (2010). Effect of systolic blood pressure reduction on hematoma expansion, perihematomal edema, and 3-month outcome among patients with intracerebral hemorrhage: results from the antihypertensive treatment of acute cerebral hemorrhage study.. Arch Neurol.

